# Contradiction between amide‐CEST signal and pH in breast cancer explained with metabolic MRI

**DOI:** 10.1002/nbm.4110

**Published:** 2019-05-28

**Authors:** Erwin Krikken, Wybe J.M. van der Kemp, Vitaliy Khlebnikov, Thijs van Dalen, Maartje Los, Hanneke W.M. van Laarhoven, Peter R. Luijten, Maurice A.A.J. van den Bosch, Dennis W.J. Klomp, Jannie P. Wijnen

**Affiliations:** ^1^ Department of Radiology University Medical Center Utrecht Utrecht The Netherlands; ^2^ Department of Surgery Diakonessenhuis Utrecht The Netherlands; ^3^ Department of Medical Oncology St. Antonius Ziekenhuis Nieuwegein/Utrecht The Netherlands; ^4^ Department of Medical Oncology, Academic Medical Centre Amsterdam Cancer Center Amsterdam Amsterdam The Netherlands; ^5^ Department of Radiology Onze Lieve Vrouwe Gasthuis Amsterdam The Netherlands

**Keywords:** APT CEST, breast cancer, 7 T MRI, 31P‐MRSI

## Abstract

**Purpose:**

Metabolic MRI is a noninvasive technique that can give new insights into understanding cancer metabolism and finding biomarkers to evaluate or monitor treatment plans. Using this technique, a previous study has shown an increase in pH during neoadjuvant chemotherapy (NAC) treatment, while recent observation in a different study showed a reduced amide proton transfer (APT) signal during NAC treatment (negative relation). These findings are counterintuitive, given the known intrinsic positive relation of APT signal to pH.

**Methods:**

In this study we combined APT MRI and ^31^P‐MRSI measurements to unravel the relation between the APT signal and pH in breast cancer. Twenty‐two breast cancer patients were scanned with a 7 T MRI before and after the first cycle of NAC treatment. pH was determined by the chemical shift of inorganic phosphate (Pi).

**Results:**

While APT signals have a positive relation to pH and amide content, we observed a direct negative linear correlation between APT signals and pH in breast tumors in vivo.

**Conclusions:**

As differentiation of cancer stages was confirmed by observation of a linear correlation between cell proliferation marker PE/Pi (phosphoethanolamine over inorganic phosphate) and pH in the tumor, our data demonstrates that the concentration of mobile proteins likely supersedes the contribution of the exchange rate to the APT signal.

**Abbreviations used**
APT: amide proton transfer
ATP: adenosinetriphosphate
CEST: chemical exchange saturation transfer
ER: estrogen receptor
FID: free induction decay
GPC: glycerophosphocholine
GPE: glycerophosphoethanolamine
GPtC: glycerophosphatidylcholine
GPtE: glycerophosphatidylethanolamine
HER2: human epidermal growth factor receptor
NAC: neoadjuvant chemotherapy
NOE: nuclear Overhauser effect;
PC: phosphocholine
PDE: phosphodiesters
PE: phosphoethanolamine
pHe: extracellular pH
pHi: intracellular pH
Pi: inorganic phosphate
PME: phosphomonoesters
PR: progesterone receptor
RF: radiofrequency
ROI: region of interest

## 1 BACKGROUND

Metabolism in cancer is widely investigated by the use of magnetic resonance imaging (MRI). Metabolic MRI is a noninvasive technique that can give new insights into understanding cancer metabolism and potentially provide biomarkers to evaluate or monitor treatment plans. Among the different techniques, a method based on chemical exchange saturation transfer (CEST)^1^ has attracted great interest recently.^2^, ^3^, ^4^, ^5^, ^6^, ^7^, ^8^ This MRI method is a powerful and sensitive technique in which low concentration solutes can be visualized through the water signal. The contrast depends on the exchange rate of saturated mobile protons to the bulk water resonance. This enables indirect imaging of endogenous molecules containing these mobile protons, such as amides (proteins and peptides), a process which is called amide proton transfer (APT). Several studies have explored the use of different CEST approaches for treatment monitoring in cancer patients such as chemotherapy,^9^, ^10^, ^11^, ^12^, ^13^, ^14^, ^15^, ^16^ radiation therapy,^17^ oncolytic virus therapy,^18^ radiosurgery^19^ and antibiotic treatment.^20^

In theory,^21^ the measured amide signal is primarily related to the concentration of mobile amide protons, the exchange rate (dependent on pH), duration of saturation pulse, and T_1_ relaxation of water. The chemical exchange rate of amide protons is base‐catalyzed.^6^ Therefore, an increase of pH results in an increase of the exchange rate and consequently causes an increase in APT signal.

All healthy cellular functioning highly depends on a strict acid–base balance (pH homeostasis). This delicate balance is influenced by many metabolic processes such as proton production, proton transportations, chemical buffering, and vascular removal of waste products. Malignant cells show a pronounced increase in metabolic processes resulting in excessive production of protons.^22^ To adapt to this intracellular acidity, the number and functions of proton‐exporting mechanisms are increased.^22^ This adaptation keeps intracellular pH (pHi) at normal or slightly alkaline levels, which favors protein synthesis and mitosis, while the extracellular pH (pHe) decreases due to the acidity from the exported protons.^23^ There are many potential pH regulators involved in this process including: Na^+^/HCO_3_^−^ co‐transporters, Na^+^/H^+^ exchangers, monocarboxylate transporters, the vacuolar ATPase, carbonic anhydrase, anion exchangers, the Cl^−^/HCO_3_^−^ exchangers, and ATP synthase.^24^, ^25^

Phosphorous magnetic resonance spectroscopy (^31^P‐MRSI) provides a noninvasive technique to measure the pHi of tumor cells. The resonant frequency of inorganic phosphate (Pi) is pH‐dependent^26^ and, in many tissues, the majority of the Pi resonance is intracellular (~ 85%).^27^ As such, pHi can be measured by ^31^P‐MRSI by calculating the chemical shift difference between Pi and a pH‐independent reference peak. Moreover, with ^31^P‐MRSI, cell proliferation biomarkers, the phoshomonoesters (PME) phosphoethanolamine (PE) and phosphocholine (PC) can be detected, providing a direct indication of cytotoxicity.^28^, ^29^ Also, the phosphodiesters (PDE) glycerophosphocholine (GPC) and glycerophosphoethanolamine (GPE) can be measured. The PME/PDE ratio is related to mitotic count and therefore indirectly to the tumor grade.^30^ This technique has been used in a previous study^31^ to show the feasibility of monitoring membrane metabolism during NAC treatment. The authors also showed that the pH was increased by 0.19 units after the completion of NAC treatment. In another study,^32^ reduced APT signal during NAC treatment was observed. These findings are counterintuitive, if one interprets the data solely in terms of the pH dependence of the chemical exchange rate of amide protons, which is base‐catalyzed. Therefore, an increase in pH results in an increased exchange rate, causing an increase in APT signal.

In this study, we combined both APT‐MRI and ^31^P‐MRSI in breast cancer patients to better comprehend the relation between APT signal and pH. APT‐MRI and ^31^P‐MRSI were acquired in breast cancer patients receiving NAC treatment before and after the first cycle of NAC using 7 T MRI.

## 2 MATERIALS AND METHODS

### 2.1 Subjects

This MRI study was performed in accordance with the guidelines of the University Medical Center Utrecht ethics committee (trialregister.nl: NTR4980). Twenty‐two breast cancer patients (aged 36–64 years, mean 47 years) gave informed consent to participate in this study. The patients were selected for being treated with NAC, and were examined before and after the first cycle of NAC (at approximately three week intervals). Table 1 summarizes the demographics and tumor characteristics of these patients.

**Table 1:** Demographics, tumor characteristics and pathological response of breast cancer patients undergoing neoadjuvant chemotherapy

Patient	Age (years)	Treatment regime	ER	PR	HER2/neu	TNM
1	58	4 x AC – 4 x docetaxel	+	−	+	T2N1M0
2	55	3 x FEC – 3 x docetaxel	+	+	−	T2N0M0
3	58	3 x FEC – 3 x docetaxel	+	+	−	T2N1M0
4	38	3 x FEC – 3 x docetaxel	+	−	−	T3N2M0
5	42	4 x AC – 4 x docetaxel	−	−	−	T2N0M0
6	38	4 x AC – 12 x paclitaxel	−	−	−	T2N3M0
7	36	4 x AC – 4 x docetaxel	+	+	−	T2N1M0
8	47	4 x AC – 4 x docetaxel	−	−	+	T2N0M0
9	42	4 x AC – 4 x docetaxel	−	−	+	T2N0M0
10	43	4 x AC – 4 x docetaxel	−	−	−	T2N0M0
11	40	4 x AC – 12 x paclitaxel	+	+	+	T1N0M0
12	41	4 x AC – 12 x paclitaxel	−	−	+	T2N1M0
13	55	4 x AC – 12 x paclitaxel	−	−	−	T2N0M0
14	53	4 x AC – 12 x paclitaxel	+	−	−	T2N1M0
15	45	4 x AC – 12 x paclitaxel	−	−	−	T2N1M0
16	48	4 x AC – 12 x paclitaxel	−	−	+	T2N0M0
17	53	6 x docetaxel ‐ AC	−	−	−	T2N0M0
18	61	6 x docetaxel ‐ AC	+	+	−	T2N1M0
19	34	6 x docetaxel ‐ AC	−	−	−	T2N0M0
20	54	6 x docetaxel ‐ AC	+	+	−	T2N1M0
21	33	6 x docetaxel ‐ AC	−	−	−	T2N1M0
22	51	6 x docetaxel ‐ AC	+	+	−	T2N0M0

AC, adriamycin and cyclophosphamide; ER, estrogen receptor; FEC, 5‐fluorouracil, epirubicin and cyclophosphamide; HER2/neu, human epidermal growth factor receptor 2; PR, progesterone receptor; TNM stage, classification of malignant tumors (tumor, nodes, metastasis).

### 2.2 Acquisition

All patients were scanned in a prone position on a 7 T MR system (Philips, Best, The Netherlands). CEST was acquired using a 26‐channel bilateral breast ^1^H transceiver coil (MR Coils, Zaltbommel, The Netherlands) and the ^31^P‐MRSI with a home‐built two‐channel unilateral ^1^H/^31^P dual‐tuned transceiver coil. Therefore, the patient was repositioned between the two measurements. Third order image‐based B_0_ shimming was performed with least square error optimization using a 3D B_0_ map followed by manual segmentation of the breasts.^33^

#### 2.2.1 Apt‐MRI

For APT‐MRI, 33 frequency offsets were acquired unevenly distributed over the frequencies from −1 ppm to 33 ppm relative to the water resonance; more offsets were obtained around the frequency of the amide peak (3.5 ppm) and the water peak (0.0 ppm) for better fitting of these resonances. The frequency offsets associated with the nuclear Overhauser effect (NOE) were not included due to signal distortions by unsuppressed lipid resonances. A saturation train of four seconds (20 sinc‐Gauss RF pulses, pulse duration = 100 ms, inter‐pulse delay = 100 ms, peak amplitude B_1 ≈_ 2 μT, duty cycle of 50%, average nominal B_1_ ≈ 0.9 μT) was followed by a gradient‐echo readout,^34^ and a short one‐to‐one spectral‐spatial RF pulse was used for fat suppression (TE = 1.4 ms, TR = 2.6 ms, flip angle = 1.2°, FOV = 150 x 320 x 100 mm^3^ [FH x RL x AP], nominal resolution = 2.3 x 3.0 x 6.8 mm^3^, 29 slices). A total of two shots with an interval of 4.48 seconds (nine seconds per frequency offset) and a 4‐fold acceleration in the right–left direction resulted in a total scan time of four minutes 55 seconds.

#### 2.2.2 ^31^P‐MRSI

The scan session consisted of a fat‐suppressed T_1_‐weighted 3D MRI (TE = 2 ms; TR = 4 ms; flip angle = 10°; FOV = 160 x 160 x 160 mm^3^; isotropic resolution of 1.0 mm^3^) for locating the tumor. ^31^P‐MRSI was obtained using the AMESING sequence^35^ (ΔTE = 45 ms, TR = 6 s, FOV = 160 x 160 x 160 mm^3^, nominal resolution = 2 x 2 x 2 cm^3^, BW = 8200 Hz, sampling matrix size = 256), where one FID and five full echoes were acquired in a total scan time of 25 minutes 36 seconds.

### 2.3 Data analysis and pH measurement

Image processing and data analysis of the CEST data were performed with MATLAB 2014b (MathWorks, Natick, MA, USA). B_0_ correction was applied using the WASSR method.^36^ APT maps were calculated using a three‐pool Lorentzian model^37^ (free water pool, APT and MT) with the Levenberg–Marquardt algorithm (fitting parameters can be found in Table 2) using the amplitude of the fit. To determine the mean APT signal in the entire tumor volume, a region of interest (ROI) was drawn on a CEST image acquired at 5.4 ppm downfield from the water resonance.

**Table 2:** Starting points and boundaries of all‐fit parameters of the three‐pool Lorentzian fit. The chemical shift δ and FWHM Γ are given in ppm

	Start	Lower	Upper
Z_base_	0.5	0.5	1
A_water_	0.8	0	1
Γ_water_	1	0.1	2.5
δ_water_	0	‐1	1
A_MT_	0.1	0	1
Γ_MT_	5	3	100
δ_MT_	0	−0.5	0.5
A_amide_	0.1	0	1
Γ_amide_	1	1	1.5
δ_amide_	3.5	3.3	3.7

δ, chemical shift; Γ, FWHM; MT, magnetization transfer.

The hypothesis, assuming the chemical exchange is dominantly base‐catalyzed and that it is directly related to the APT signal, was calculated using: *k* = *k*_0_+[*k*_*b*_ × 10^*pH* − *pKw*^], with *k*_0_ = 26.8, *k*_*b*_ = 3.4×10^6^, and *pK*_*w*_ = 11.2.^6^, ^21^ This relationship was used to compare it with the measured data.

All MRSI data were analyzed using IDL 6.3 (Research Systems, Boulder, CO, USA), jMRUI 4.0^38^ and MATLAB 2014b. Voxel selection of the breast tumor was performed in an in‐house built program in MATLAB on the fat‐suppressed T_1_‐weighted 3D MRI. All spectra were zero‐filled to 8192 data points and apodized (15 Hz Lorentzian) in the time domain and spatially Hamming‐filtered. For the pH measurement, all spectra were aligned to α‐ATP at 7.56 ppm as α‐ATP is the most insensitive to pH of all peaks with high signal‐to‐noise ratio. pH values were calculated using the following form of the Henderson–Hasselbalch equation^39^:

$$\mathrm{pH}=pK_{A}+\log_{10}\left(\frac{\delta -\delta_{\mathrm{HA}}}{\delta_{A}-\delta }\right)$$

where pK_A_ = 6.75 is the dissociation constant of Pi, δ_HA_ = 3.27 is the chemical shift of the protonated form of Pi, δ_A_ = 5.69 is the chemical shift of the nonprotonated form of Pi, and δ is the difference in chemical shift frequency between the Pi peak and the reference α‐ATP peak, measured in parts per million (ppm).

^31^P‐MRSI is known to be used to image metabolites involved in the disturbed anabolism and catabolism of the cell membrane in breast cancer. Metabolite ratios such as PME/PDE ratio and phosphoethanolamine/inorganic phosphate (PE/Pi) ratio were related to pH and APT signal. For calculating the metabolic signal ratios for PME/PDE and PE/Pi, all spectra were frequency‐aligned to PE at 6.83 ppm and quantified using a nonlinear least‐squares algorithm (AMARES).^40^

### 2.4 Statistical analysis

Statistical analysis was performed in GraphPad Prism (GraphPad Software, San Diego, CA, USA). A linear regression was used to determine the relation between the measured APT signal, metabolic ratios and the pH before and after the first cycle of NAC treatment. The relation was considered statistically significant if *P* < 0.05. Relative changes of these parameters, as measured prior to and after the first NAC cycle, were quantified. Values less than the first quartile ‐ 1.5 x interquartile range, or greater than the third quartile +1.5 interquartile range, were determined as outliers (<[Q1–1.5 x IQR] or > [Q3 + 1.5 x IQR]).

## 3 RESULTS

The mean age of the 22 patients was 47 years (33–61 years) and each patient completed all cycles of NAC treatment (four different regimes were used; Table 1). Assessment of hormone receptor status was performed on the pretreatment core biopsy; six patients had human epidermal growth factor receptor 2 (HER2) positive tumors and 16 patients had HER2 negative tumors. Ten tumors were estrogen receptor (ER) positive, of which seven were progesterone receptor (PR) positive. Eight tumors were triple negative.

**Figure 1:**
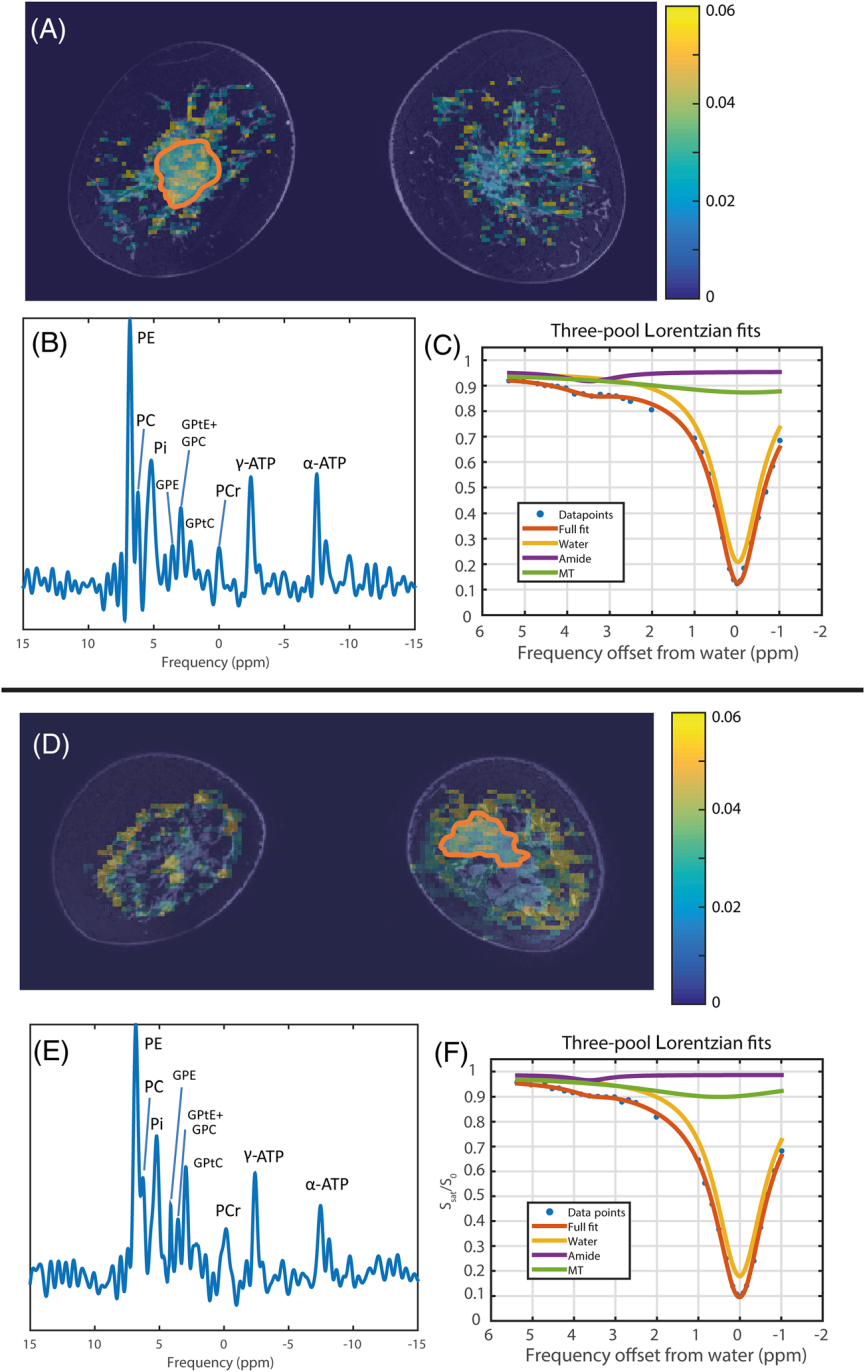
Example of acquired and calculated data (patients 13, upper half, and 14, lower half from Table 1 before the start of NAC treatment). (A,D) coronal slices of the calculated APT maps with the ROIs of the tumor (orange) overlaid on top of a fat suppressed T_1_‐weighted 3D FFE (TR = 7.1 ms; TE = 3.2 ms; flip angle = 8°; resolution of 0.7 mm^3^; SENSE 4 x 2 [RL x FH], 1–4–6‐4‐1 spectral spatial RF pulse for fat suppression). Note that both ^31^P‐MRS and CEST analysis were performed on the entire tumor volume; only one coronal slice is shown here. (B,E) corresponding ^31^P‐MRSI spectra originating from the ROI where nine metabolites are visible. (C,F) three‐pool Lorentzian fit of the Z‐spectra of water (yellow line), magnetization transfer effect (MT; green line), amide proton transfer (APT; purple line) and the full fit consisting of the three fits (orange)

An example of the analysis of the acquired data of a patient (patient 13 from Table 1 before the start of NAC treatment) is shown in Figure 1. For analysis of ^31^P‐MRSI, one voxel containing the tumor was selected (Figure 1A) and nine metabolites were fitted (Figure 1B). The metabolic ratios of metabolites involved in membrane metabolism (PME/PDE and PE/Pi) and the pH (based on the chemical shift between Pi and α‐ATP) were calculated. From the APT‐MRI, the mean APT signal was determined inside the tumor volume (Figure 1C) based on the three‐pool Lorentzian fit.

**Figure 2:**
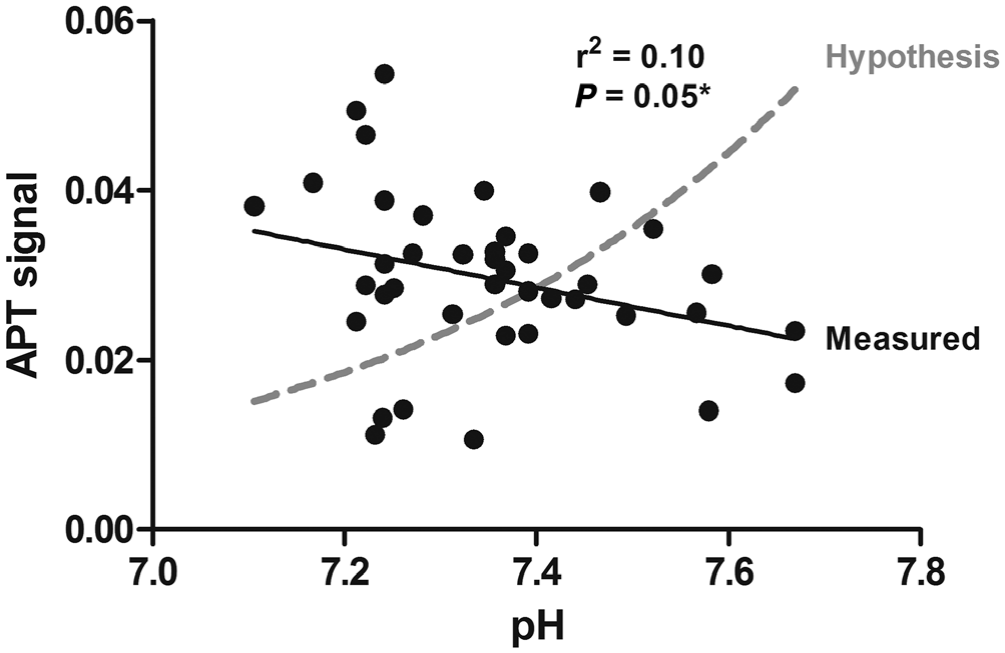
Relation between APT signal and pH measured by CEST and ^31^P‐MRS (based on the chemical shift between pi and α‐ATP) for all patients before and after the first cycle of NAC treatment. The hypothesis (dashed gray line) was calculated based on Sun and Sorensen^6^ using k = k_0_+[k_b_ × 10^pH − pKw^], with k_0_ = 26.8, k_b_ = 3.4×10^6^, and pK_w_ = 11.2 and normalized to the measured data. The linear regression is shown by the solid black line which is already statistically significant without correcting for known (Sun and Sorensen) pH effects (P < 0.05). Note that the measured data is contradicting the hypothesis; a decreasing APT‐CEST with increasing pH opposed to the hypothesis; *, Statistically significant

**Figure 3:**
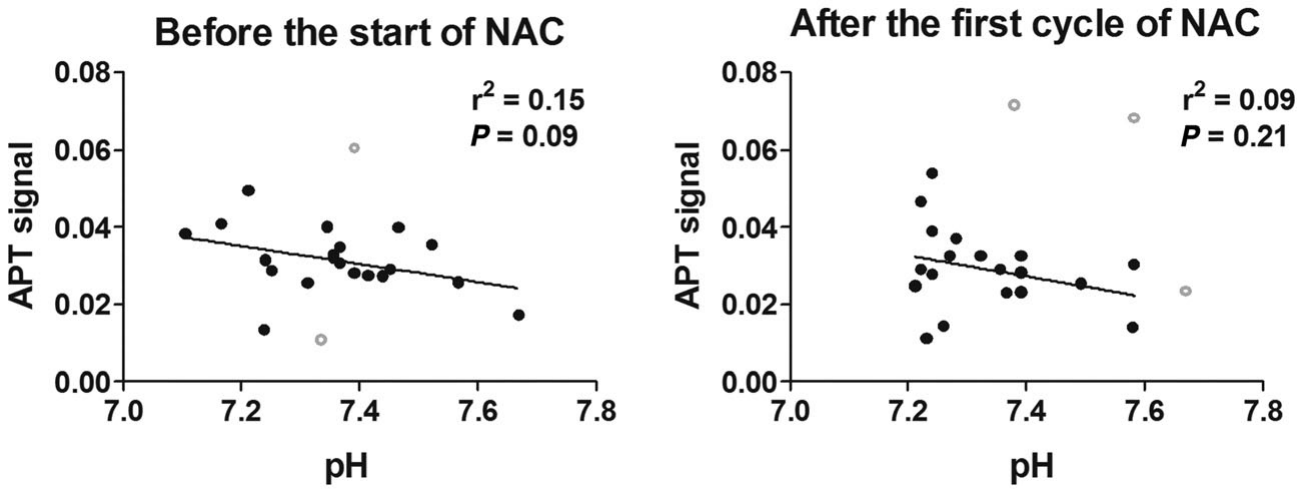
Relation of APT signal with pH (left) before and (right) after the first cycle of NAC treatment. Separating the data in these two groups resulted in a linear regression which was no longer statistically significant for both. The outliers are shown in gray

We found a statistically significant correlation between APT‐CEST and pH when combining all data from all patients (Figure 2). This correlation was, however, in the opposite direction to that which the hypothesis proposed (dashed gray line); an increase of pH showed a decrease in APT‐CEST signal. When splitting the group into before and after the first cycle of NAC treatment, the linear regressions were still in the opposite direction compared with the hypothesis, yet no longer statistically significant (Figure 3).

**Figure 4:**
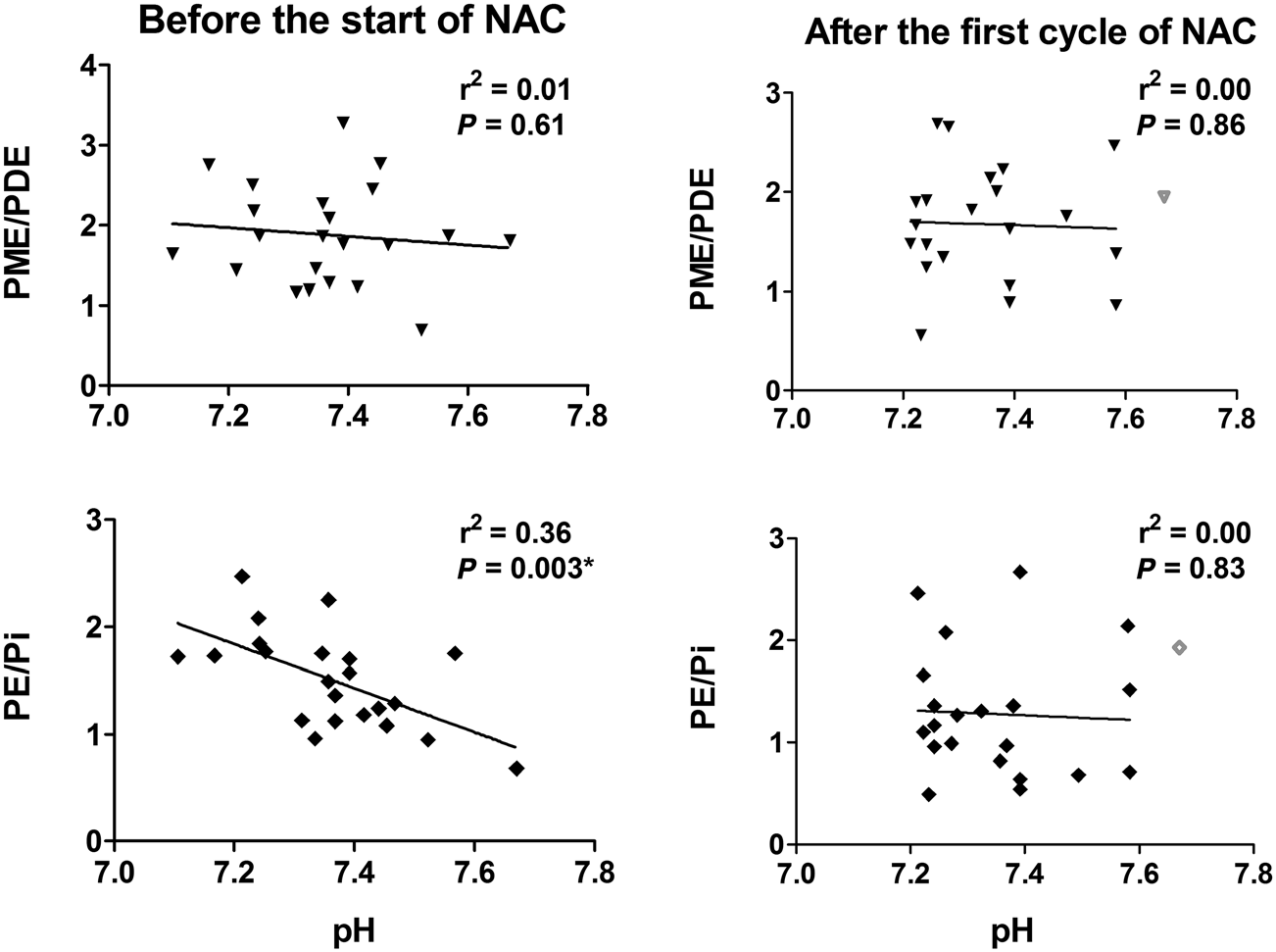
Relation of PME/PDE and PE/pi with pH (left) before and (right) after the first cycle of NAC treatment. The linear regressions are shown as black solid lines. The linear regression between PE/pi and pH before the start of NAC treatment was statistically significant. The outliers are shown in gray. *, Statistically significant

The PME/PDE ratio, known to be involved with membrane anabolism and catabolism, showed no significant correlation with pH (Figure 4). A significant negative correlation between PE/Pi ratio and pH was found prior to NAC treatment. After the first cycle of NAC treatment, the correlation was no longer statistically significant.

An absolute mean shift of 0.1 units of pH was observed after the first cycle of NAC treatment with a maximum of 0.3 units of pH in individual patients. When averaged over all patients, the tumor tissue had a slightly alkaline pH of 7.4 before the start of NAC treatment and did not change after the first cycle of NAC treatment.

## 4 DISCUSSION

In this study we acquired a unique dataset at 7 T consisting of the APT‐MRI and ^31^P‐MRSI of breast cancer patients receiving NAC treatment. This data enabled the investigation of the relation between APT and pH with a direct measure of pH through ^31^P‐MRSI, which has not been presented before. Based on the chemical shift between Pi and α‐ATP from the ^31^P‐MRSI spectra, we calculated the pH of all patients before and after the first cycle of NAC treatment. We showed that there was a statistically significant correlation between the APT signal and pH and between PE/Pi and pHi before the start of NAC treatment. After the first cycle of NAC treatment, these correlations appeared to be no longer statistically significant. The pHi (the Pi signal mostly originates from the cytosolic compartment^41^) remained neutral to slightly alkaline, which tumors are known to be.

We showed that proliferation measured with PE/Pi is higher with lower pHi, which suggests increased cellularity at lower pHi. This would also suggest that there will be an increased concentration of proteins, resulting in higher CEST effects at lower pH, mainly due to the concentration of proteins and peptides. This was confirmed by the APT measurements during this study. However, this is in contradiction to the naïve interpretation that APT must be smaller at lower pH due to the lower base‐catalyzed amide proton exchange rate, but hints at the finding that, for the APT signal, concentration effects outweigh direct pH effects. The measured APT signal depends on physiological parameters and sequence parameters. First, the physiological parameters are discussed.

As mentioned before, APT signal is primarily related to the concentration of mobile amide protons, the amide proton exchange rate, and T_1_ relaxation of water. A change in T_1_ relaxation time due to therapy effects could influence the APT signal, as T_1_ relaxation is positively related with APT signal.^42^, ^43^ However, the APT signal varies by a factor of two between tumors, and this cannot solely be assigned to changes in T_1_ relaxation time, as this would mean differences in tumor T_1_ of 50%,^44^ which is unrealistic. Therefore, we assumed that the expected effect size of T_1_ relaxation on the APT signal is much less in comparison with the concentration of mobile amide protons and the exchange rate.

In normal tissue, it is expected that the extracellular compartment contains a low concentration of mobile amides. Therefore, APT signal in normal tissue most likely originates from proteins and peptides in the intracellular space. In tumor tissue, however, the extracellular space could contain increased concentrations of mobile proteins and peptides as a result of accumulation of blood‐borne proteins, such as albumin, due to perforated blood vessels.^45^ Therefore, both intracellular and extracellular compartments are likely to contribute to APT signal originating from tumor tissue. Intracellular pH in tumor tissue did not change much (slightly alkaline, mean pH of 7.4) and so APT signal change, originating from the intracellular compartment, assuming that T_1_ relaxation will have the least effect (see above), reflects a change in protein content. Extracellular pH in tumors is acidic and APT‐MRI, originating from the extracellular compartment, reflects a change in protein content and pH. Only a small part of the pH (~ 15%) that is determined by ^31^P‐MRS originates from the extracellular compartment and therefore it is difficult to rule out parameters affecting the APT signal originating from the extracellular compartment. However, this data does suggest that the concentration of the mobile amide protons is the main contributor to the observed APT signal.

Considering the main influence of the concentration of the mobile amide protons, the dependency of the APT signal on the mobile amide protons is much stronger than its contra‐related dependency to the exchange rate. A recent study in a rat model of brain metastasis determined that the proportion of APT signal originating from changes in protein concentration was approximately 66%, with the remaining 34% originating from changes in tumor pH.^46^

It has been shown that NOE signals from aromatic protons, in a range of +1 to +5 ppm from water, affect the quantification of APT effects.^47^ These aromatic NOE signals also originate from proteins, yet are insensitive to pH. If these signals decrease due to therapy effects, they could surpass the effect of the actual amides, resulting in this inverse pH dependency. It could also be that overlapping CEST signals from different exchanging sites influence the APT signal. It has been shown that fast‐exchanging amines can have an inverse pH dependency in animals^48^ and homogenates.^49^ However, the nominal B_1_ in this study was 2 μT (sinc‐Gaussian pulses) with a duty cycle of 50%. This means that the average nominal B_1_ is less than 0.9 μT. Also, the relative B_1_ in the tumor area is approximately 50–60% (see below), resulting in approximately 0.5 μT average effective B_1_ in the tumor. Considering this B_1_, the main contributor to the measured signal is APT (approximately 90%),^50^ which makes the contribution of fast‐exchanging protons highly unlikely. A more likely explanation for the decrease of APT with increasing pH would be the decreasing cellularity (PE/Pi) with pH. A low cellularity hints at a lowered concentration of proteins, which is a more probable explanation for the decreased APT signal.

A sequence‐dependent parameter affecting APT signals is B_1_ inhomogeneity. In this study, the CEST was optimized for signal generated by the slow‐exchanging amide protons, and the optimal B_1_ for detecting this exchange is approximately 1 μT.^51^ The peak B_1_ amplitude in this study was set to 2 μT to account for B_1_ loss in the hardware setup. The bilateral breast coil setup consisted of two quadrature RF coils placed in front of the breasts, which led to a decrease in B_1_, varying from 60% in the front of the breast to 50% towards the pectoral muscle. This means that the APT signal coming from tumors located near the nipple could be higher than the APT signal coming from tumors located near the pectoral muscle. Also, the level of variance in B_1_ and its effect on the APT signal cannot explain the level of observed APT signal changes, particularly when considering that the tumor location with respect to the RF coil is not expected to change substantially over one cycle of chemotherapy.

The overall calculated APT contrast could also be influenced by the degree of fat suppression. We used RF and gradient spoiling to reduce lipid artefacts. However, insufficient fat suppression in the tumor may have resulted in an underestimation of the CEST amplitude,^52^ possibly affecting the change in APT signal.

Also, the zero‐order approach used in this study is not exact, and different power levels will have different contributions of MT and water saturation, probably leading to different sized APT effects. However, all of the experiments were performed with the same parameters for the RF pulses and, therefore, this will not affect our conclusion.

We were able to perform ^31^P‐MRSI analysis for every patient. A linear relationship was found in this study between PE/Pi before NAC treatment and the pHi. PE is a key metabolite involved in the Kennedy pathway that produces phosphatidylserine in the major building block of cell membranes.^29^ Pi is involved in many metabolic pathways, including energy transfer, protein activation, and carbon and amino acid metabolic processes.^53^ Therefore, PE/Pi could be an indicator for cellularity. After the first cycle of NAC this correlation is not significant. NAC treatment has been shown to be most effective if a combination of anthracyclines and taxanes is used, causing damage to DNA and disrupting the pathways necessary to facilitate mitosis.^54^ Therefore, it is most likely that PE, Pi and pH in the tumor are all affected by the chemotherapy. However, further research is required to understand the linear relationship between PE/Pi and pH.

In conclusion, in this study we have shown that APT‐MRI and ^31^P‐MRSI provide complementary information about tumor metabolism in breast cancer patients. A linear correlation between APT signal and pH, and a linear correlation between PE/Pi and pH in the tumor were found in breast cancer patients before the start of NAC treatment. This correlation was opposite to the intrinsic relation between APT signal and pH, demonstrating that the concentration of mobile amide protons is the main contributor to the observed APT signal. In fact, when correcting for the known intrinsic relation of APT with pH, the observed concentration range of mobile amides will be even higher.
